# Supplementary material for: Total Synthesis of Loroxanthin
Source: Mar Drugs. 2022 Oct 24;20(11):658. doi: 10.3390/md20110658 (PMC9699617; doi:10.3390/md20110658)
Supplement: Supplementary file 1 [file marinedrugs-20-00658-s001.zip › marinedrugs-1980087-supplementary.pdf]

Supplementary Materials for

Total Synthesis of Loroxanthin

Yumiko Yamano [1,2,*], Mari Tanabe [2], Atsushi Shimada [2] and Akimori Wada [2]

[1] Comprehensive Education and Research Center, Kobe Pharmaceutical University, Kobe 658-8558, Japan
[2] Laboratory of Organic Chemistry for Life Science, Kobe Pharmaceutical University, Kobe 658-8558, Japan
* Correspondence: y-yamano@kobepharma-u.ac.jp

Table of Contents

Figure S1. ^{1}H-NMR spectrum ($CDCl_3$, 300 MHz) of compound **15** ---------------------------------- 3

Figure S2. ^{13}C-NMR spectrum ($CDCl_3$, 75 MHz) of compound **15** ------------------------------------3

Figure S3. ^{1}H-NMR spectrum ($CDCl_3$, 300 MHz) of compound **10** ---------------------------------- 4

Figure S4. ^{13}C-NMR spectrum ($CDCl_3$, 75 MHz) of compound **10** ------------------------------------4

Figure S5. ^{1}H-NMR spectrum ($CDCl_3$, 300 MHz) of compound **16** ---------------------------------- 5

Figure S6. ^{13}C-NMR spectrum ($CDCl_3$, 75 MHz) of compound **16** ------------------------------------5

Figure S7. ^{1}H-NMR spectrum ($CDCl_3$, 300 MHz) of compound **17** ---------------------------------- 6

Figure S8. ^{13}C-NMR spectrum ($CDCl_3$, 75 MHz) of compound **17** ------------------------------------6

Figure S9. ^{1}H-NMR spectrum ($CDCl_3$, 300 MHz) of compound **18** ---------------------------------- 7

Figure S10. ^{13}C-NMR spectrum ($CDCl_3$, 75 MHz) of compound **18** -----------------------------------7

Figure S11. ^{1}H-NMR spectrum ($CDCl_3$, 500 MHz) of compound **19** ----------------------------------8

Figure S12. ^{13}C-NMR spectrum ($CDCl_3$, 125 MHz) of compound **19** --------------------------------8

Figure S13. ^{1}H-NMR spectrum ($CDCl_3$, 500 MHz) of compound **8** ----------------------------------- 9

Figure S14. ^{13}C-NMR spectrum ($CDCl_3$, 125 MHz) of compound **8** --------------------------------- 9

Figure S15. ^{1}H-NMR spectrum ($CDCl_3$, 500 MHz) of 9*E*-isomer of compound **8** ------------------10

Figure S16. ^{13}C-NMR spectrum ($CDCl_3$, 125 MHz) of 9*E*-isomer of compound **8** -----------------10

Figure S17. ^{1}H-NMR spectrum ($CDCl_3$, 300 MHz) of compound **23** -------------------------------- 11

Figure S18. ^{13}C-NMR spectrum ($CDCl_3$, 75 MHz) of compound **23** ---------------------------------11

Figure S19. ^{1}H-NMR spectrum ($CDCl_3$, 300 MHz) of compound **24** -------------------------------- 12

Figure S20. ^{13}C-NMR spectrum ($CDCl_3$, 75 MHz) of compound **24** ---------------------------------12

Figure S21. ^{1}H-NMR spectrum ($CDCl_3$, 300 MHz) of compound **25** -------------------------------- 13

Figure S22. ^{13}C-NMR spectrum ($CDCl_3$, 75 MHz) of compound **25** ---------------------------------13

Figure S19. ^{1}H-NMR spectrum ($CDCl_3$, 500 MHz) of loroxanthin (**1**) -------------------------------14

Figure S20. ^{13}C-NMR spectrum ($CDCl_3$, 125 MHz) of loroxanthin (**1**) ------------------------------14

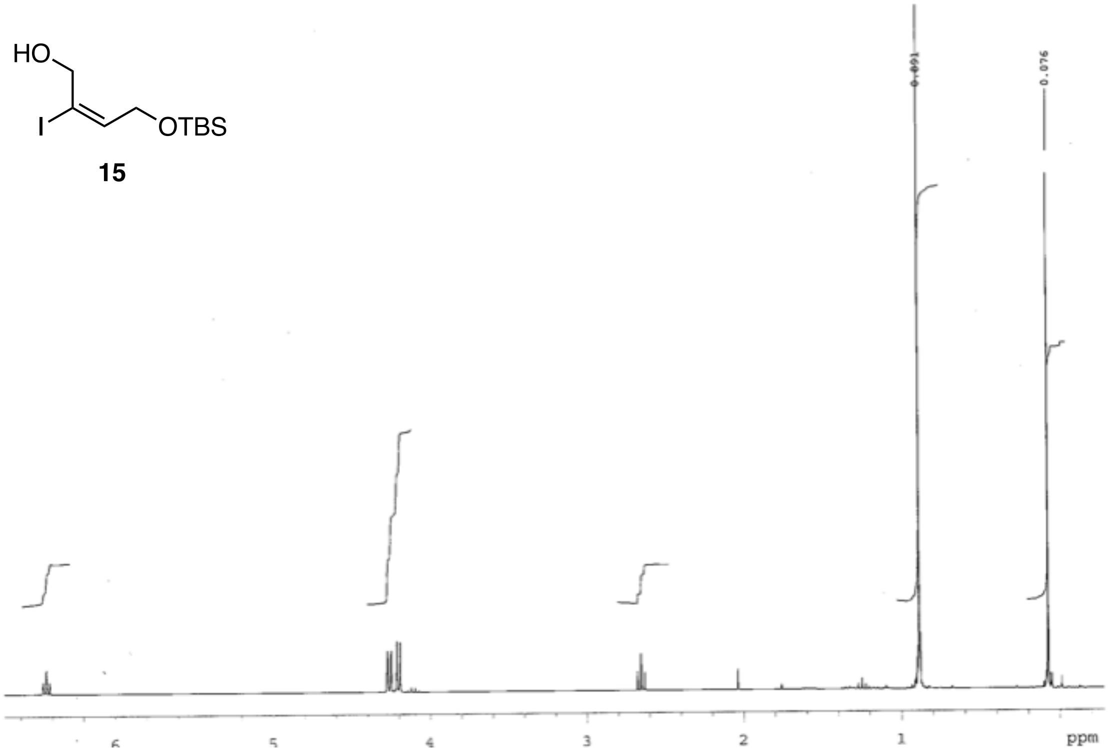

Figure S1. ^{1}H-NMR spectrum (CDCl, 300 MHz) of compound **15**

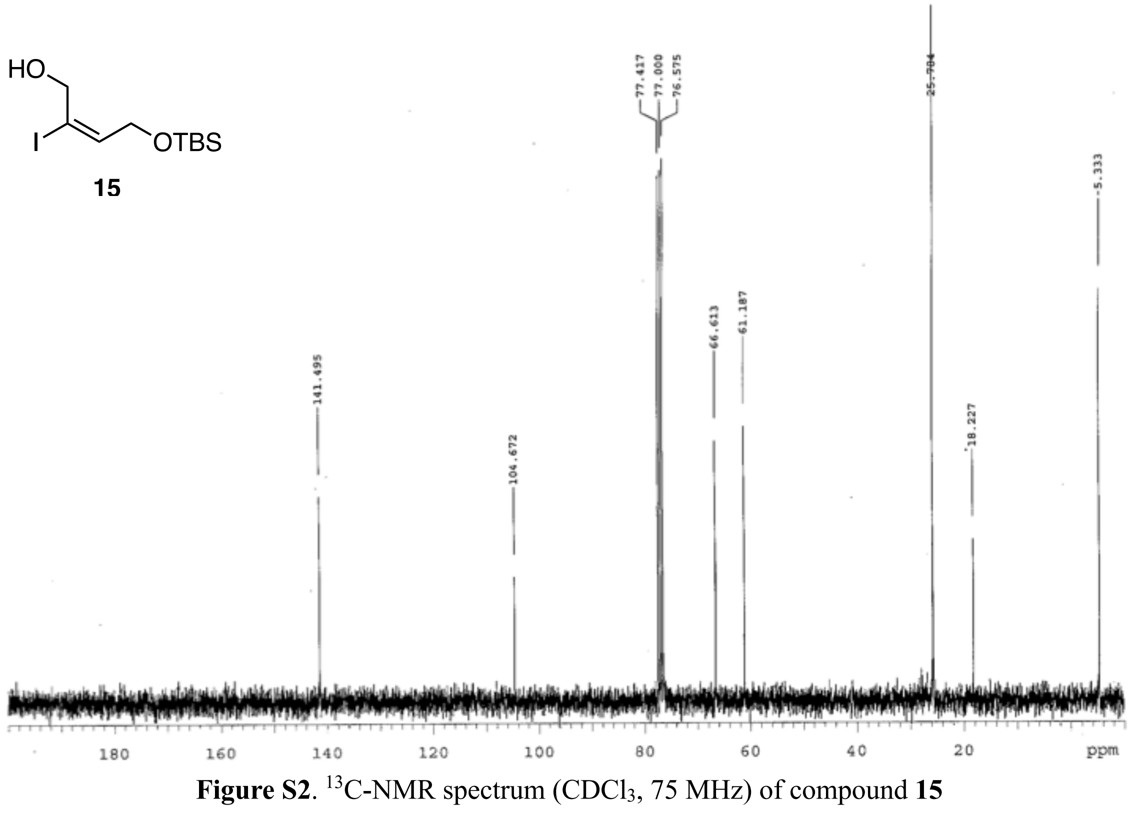

Figure S2. ^{13}C-NMR spectrum ($CDCl_3$, 75 MHz) of compound **15**

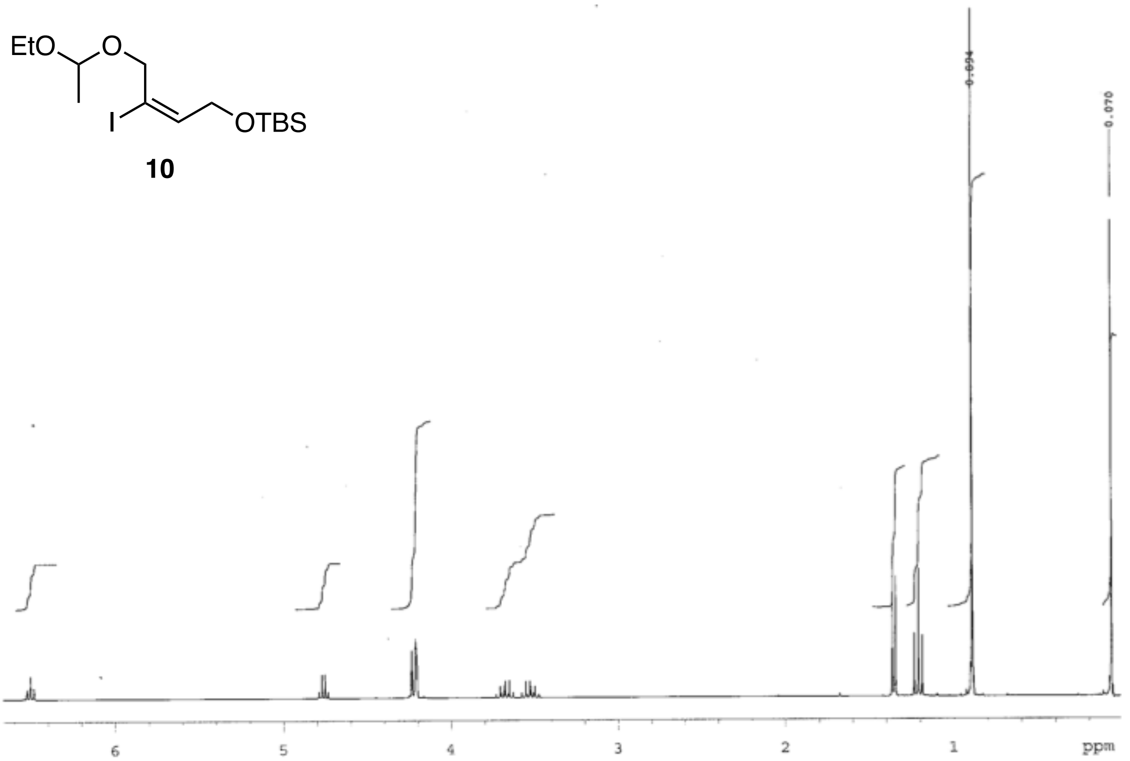

Figure S3. ^{1}H-NMR spectrum ($CDCl_3$, 300 MHz) of compound **10**

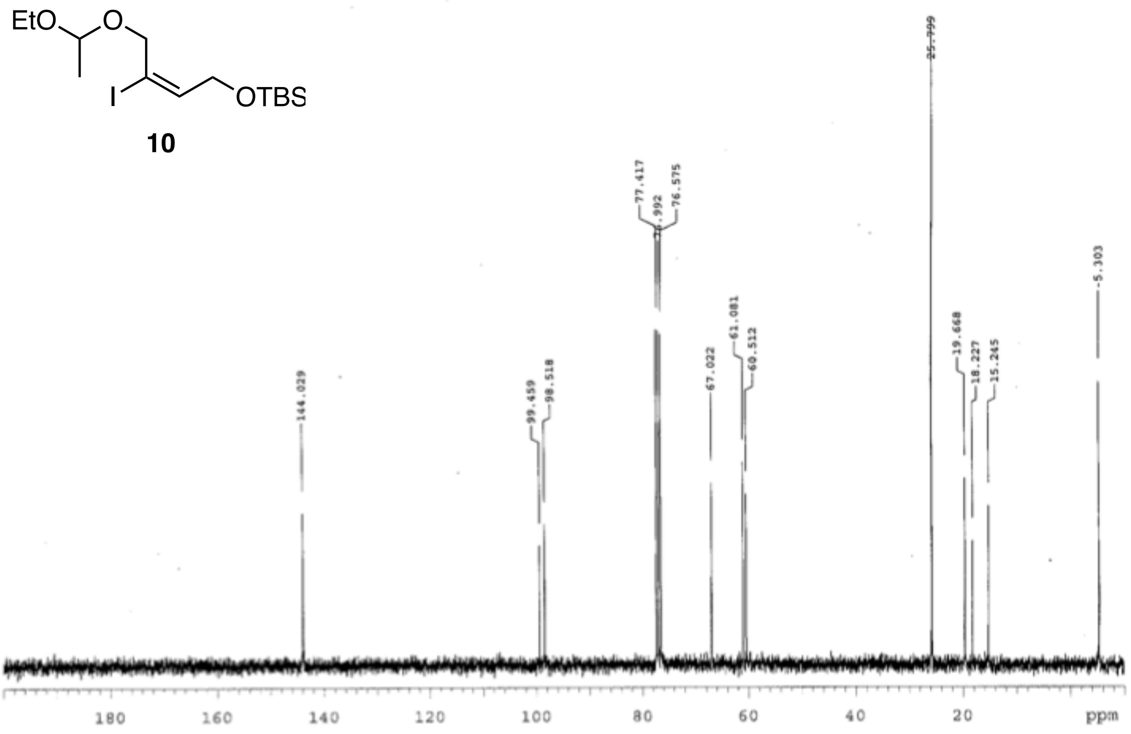

Figure S4. ^{13}C-NMR spectrum ($CDCl_3$, 75 MHz) of compound **10**

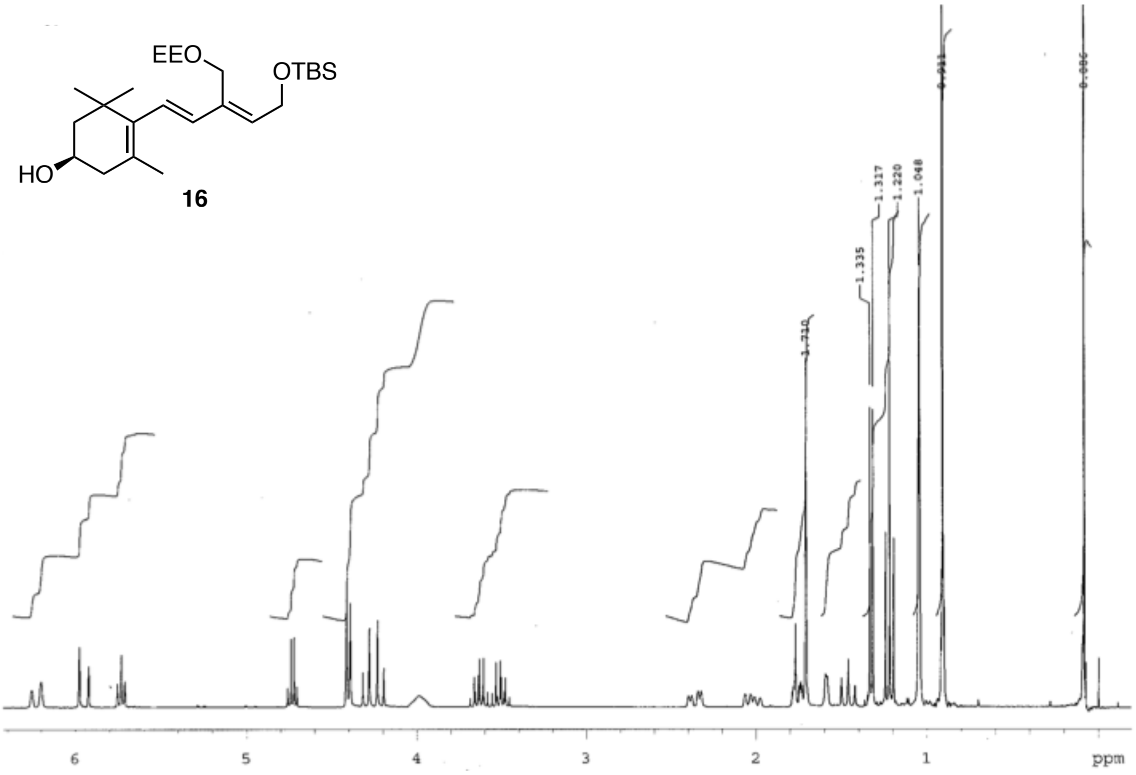

Figure S5. ^{1}H-NMR spectrum ($CDCl_3$, 300 MHz) of compound **16**

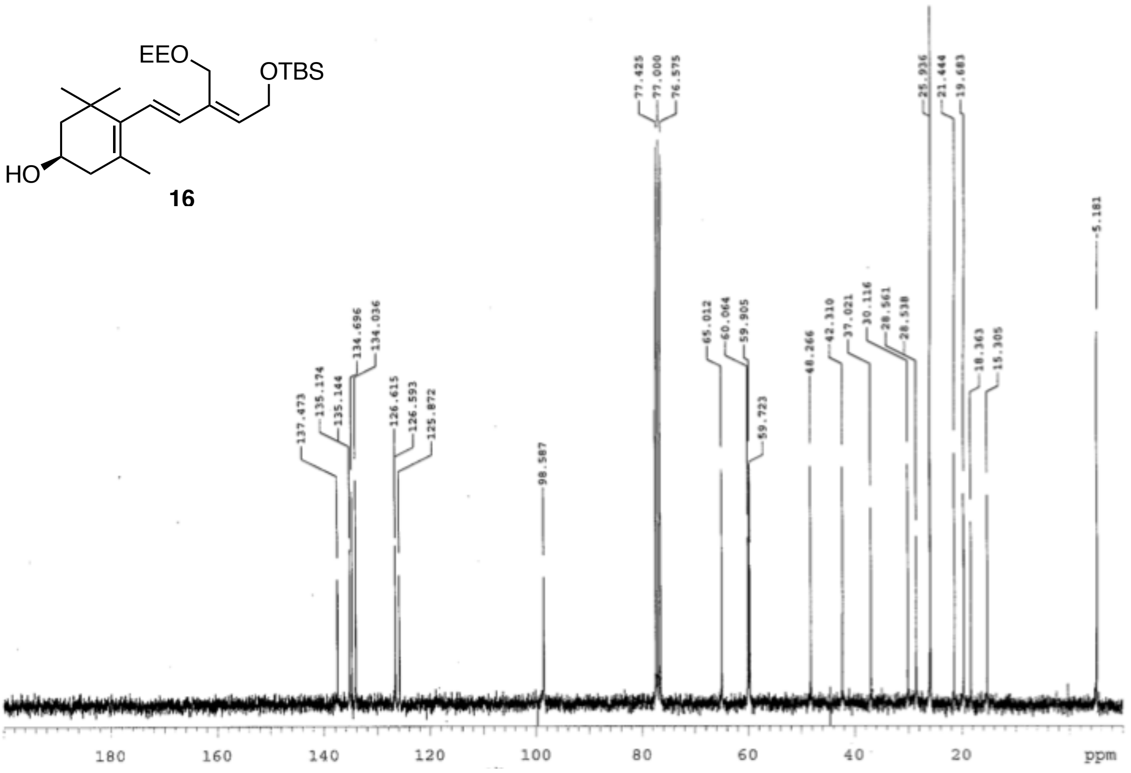

Figure S6. ^{13}C-NMR spectrum ($CDCl_3$, 75 MHz) of compound **16**

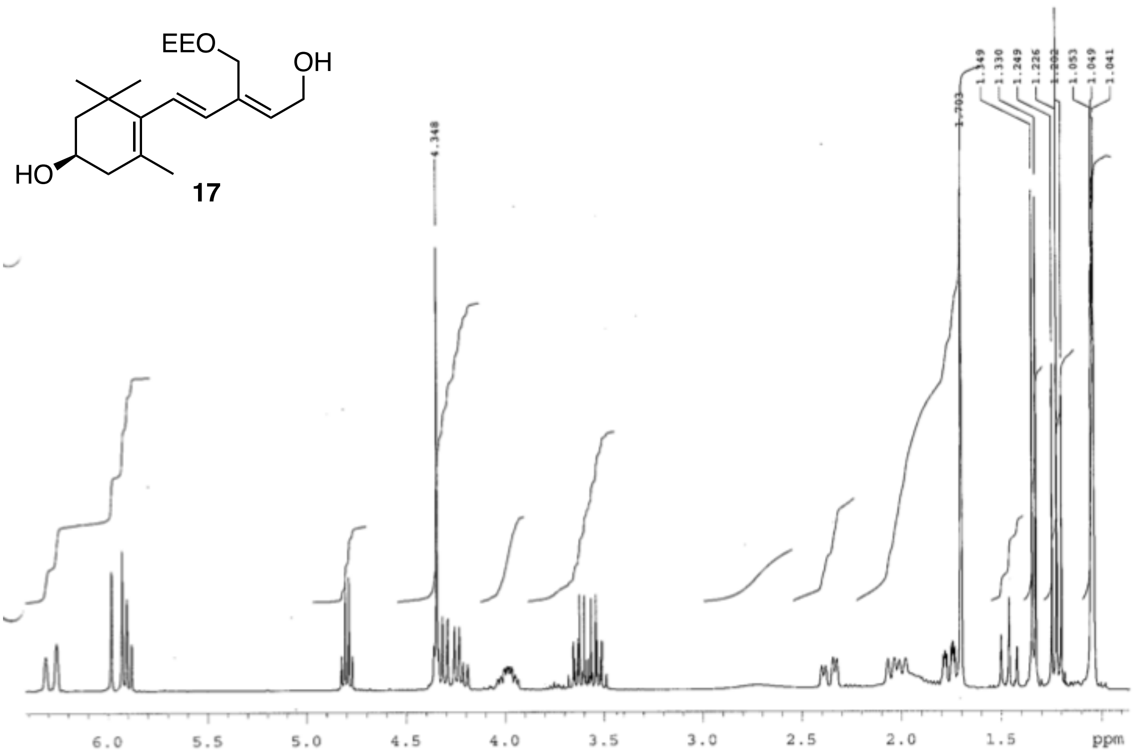

Figure S7. ^{1}H-NMR spectrum ($CDCl_3$, 300 MHz) of compound **17**

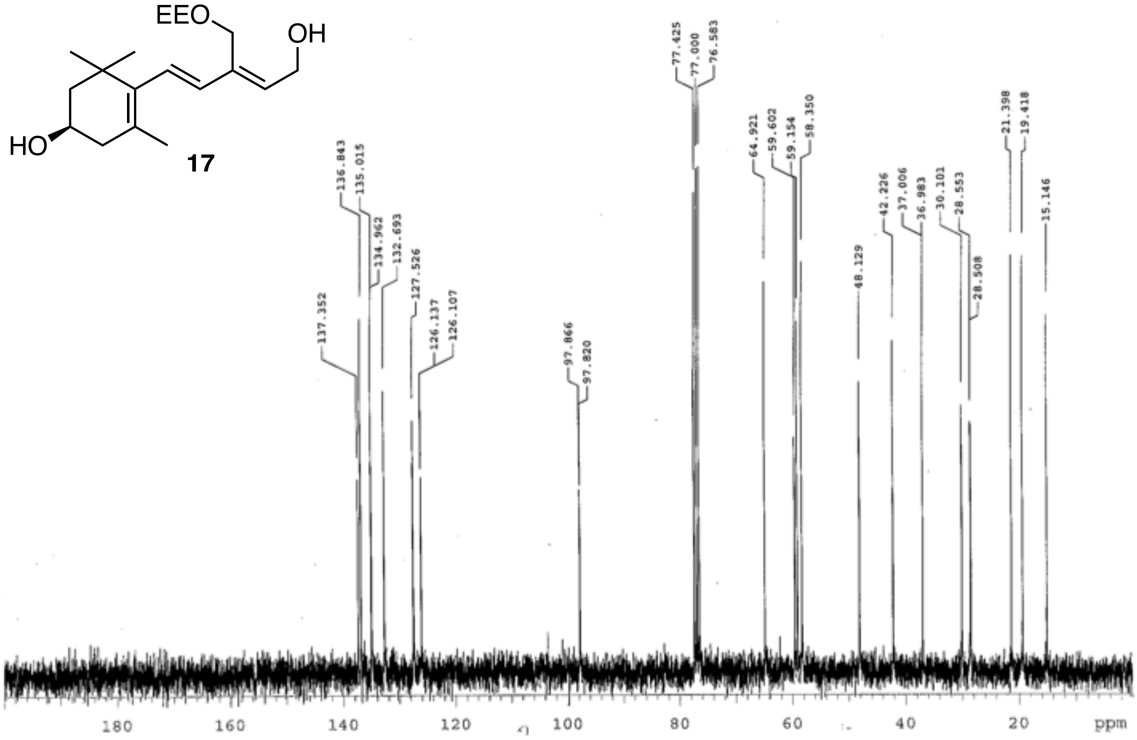

Figure S8. ^{13}C-NMR spectrum ($CDCl_3$, 75 MHz) of compound **17**

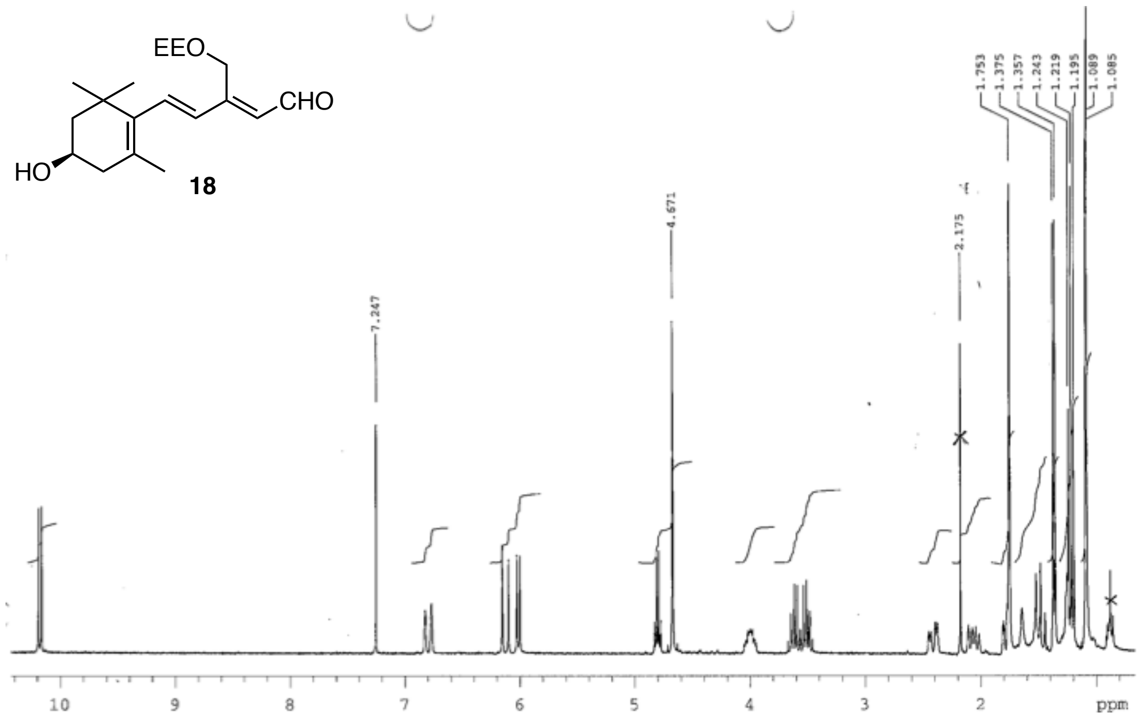

Figure S9. ^{1}H-NMR spectrum ($CDCl_3$, 300 MHz) of compound **18**

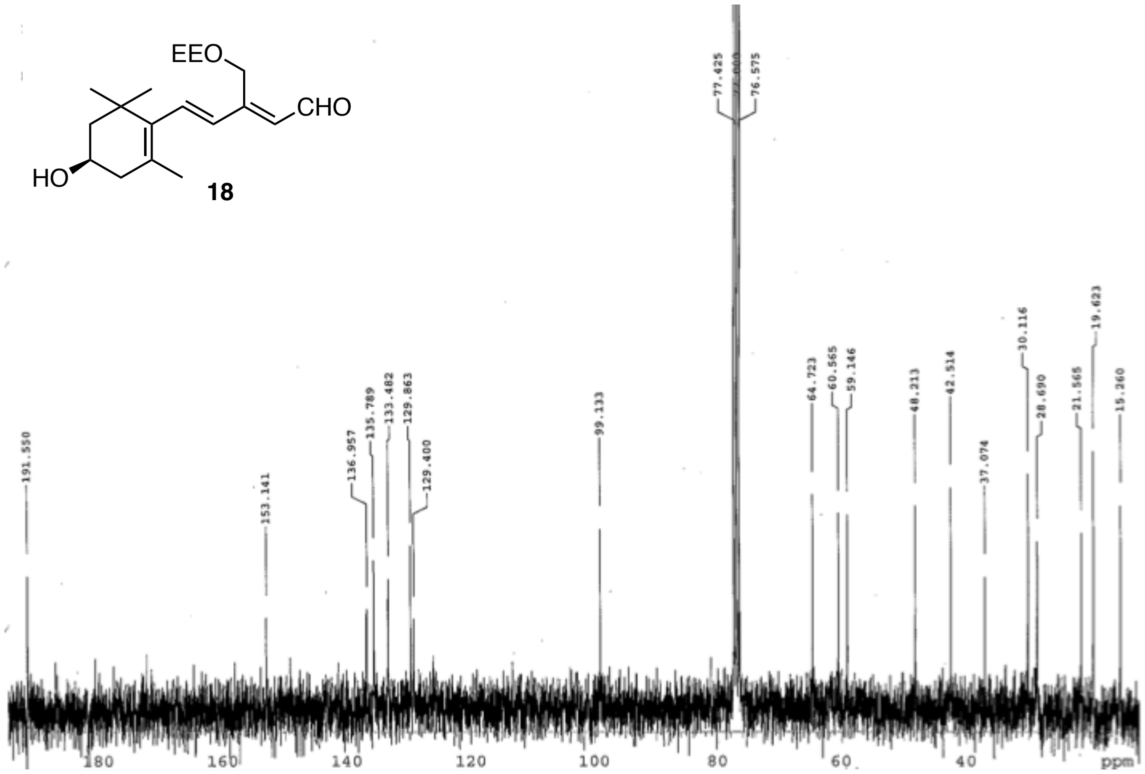

Figure S10. ^{13}C-NMR spectrum ($CDCl_3$, 75 MHz) of compound **18**

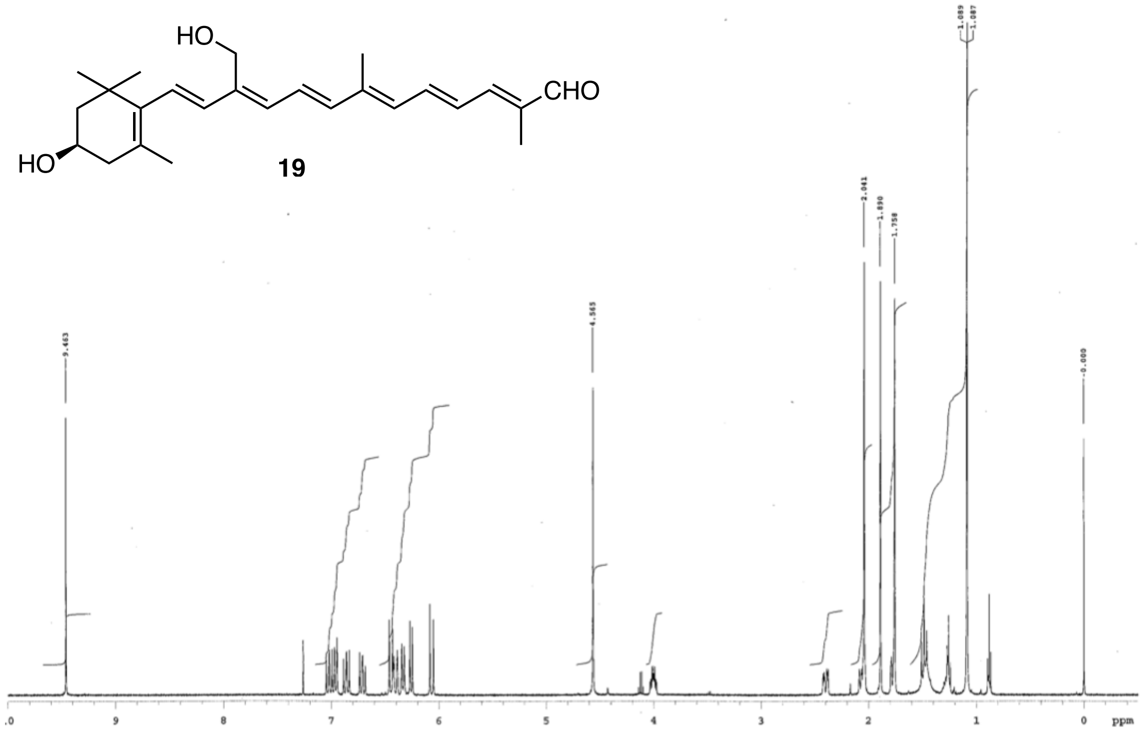

Figure S11. ^{1}H-NMR spectrum ($CDCl_3$, 500 MHz) of compound **19**

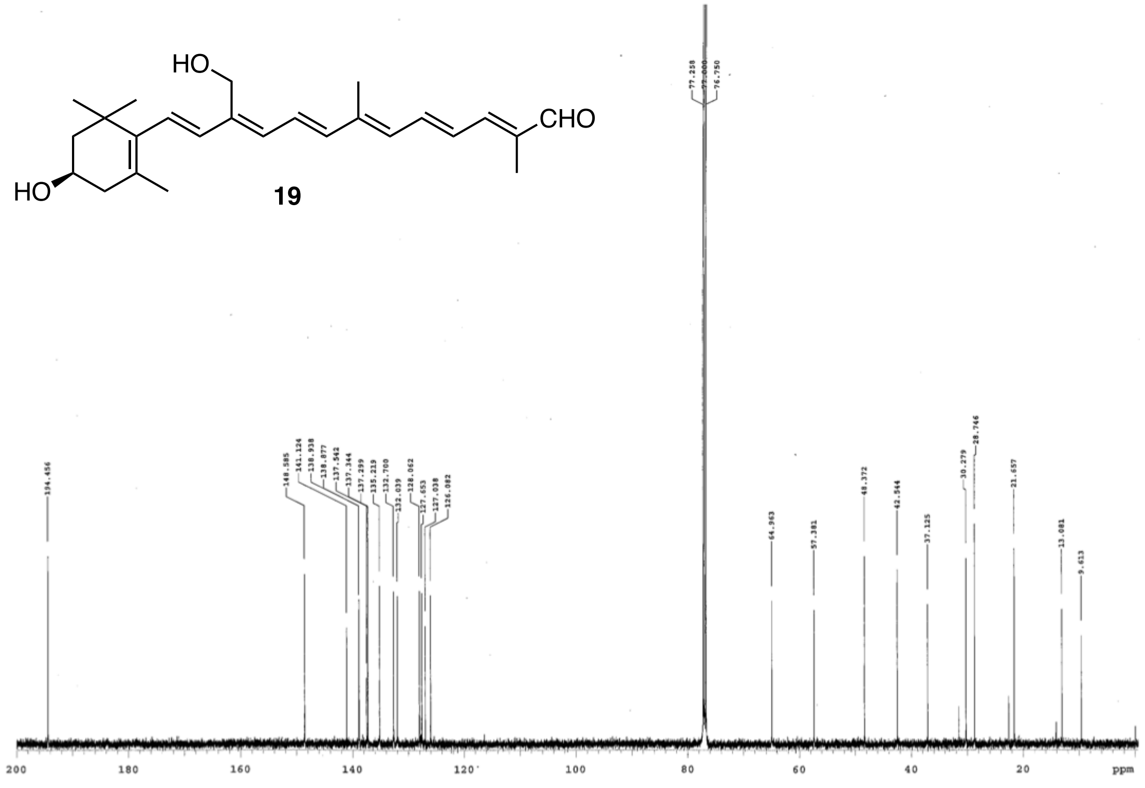

Figure S12. ^{13}C-NMR spectrum ($CDCl_3$, 125 MHz) of compound **19**

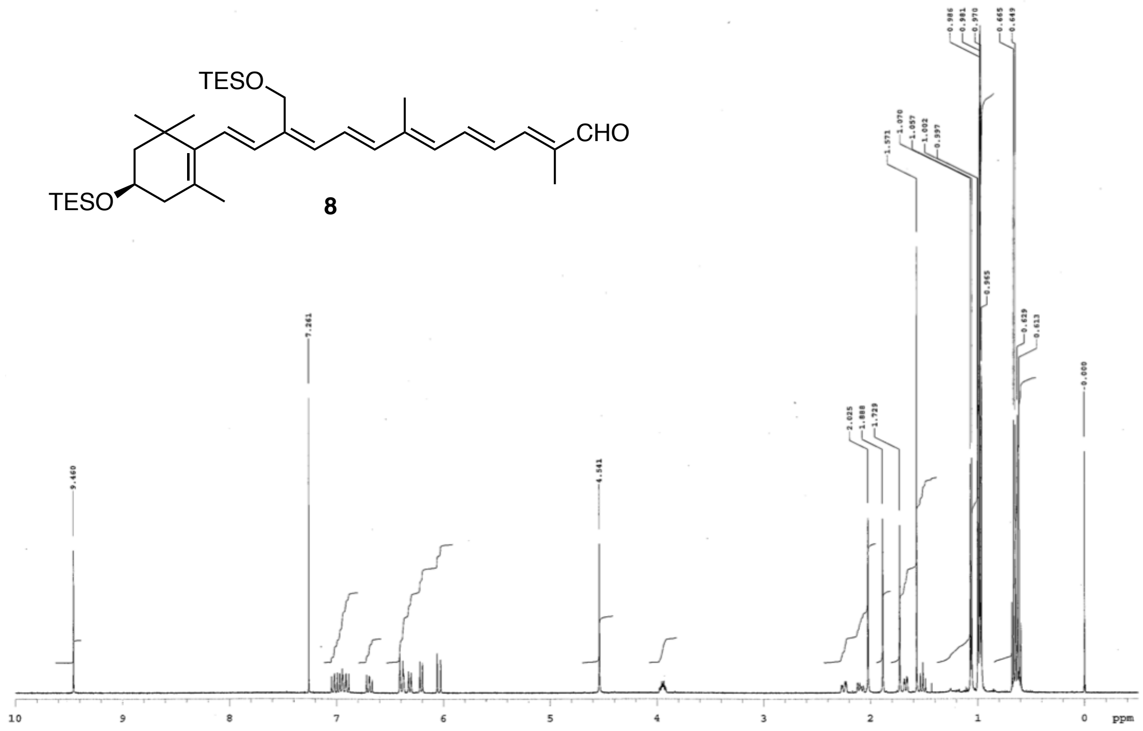

Figure S13. ^{1}H-NMR spectrum ($CDCl_3$, 500 MHz) of compound **8**

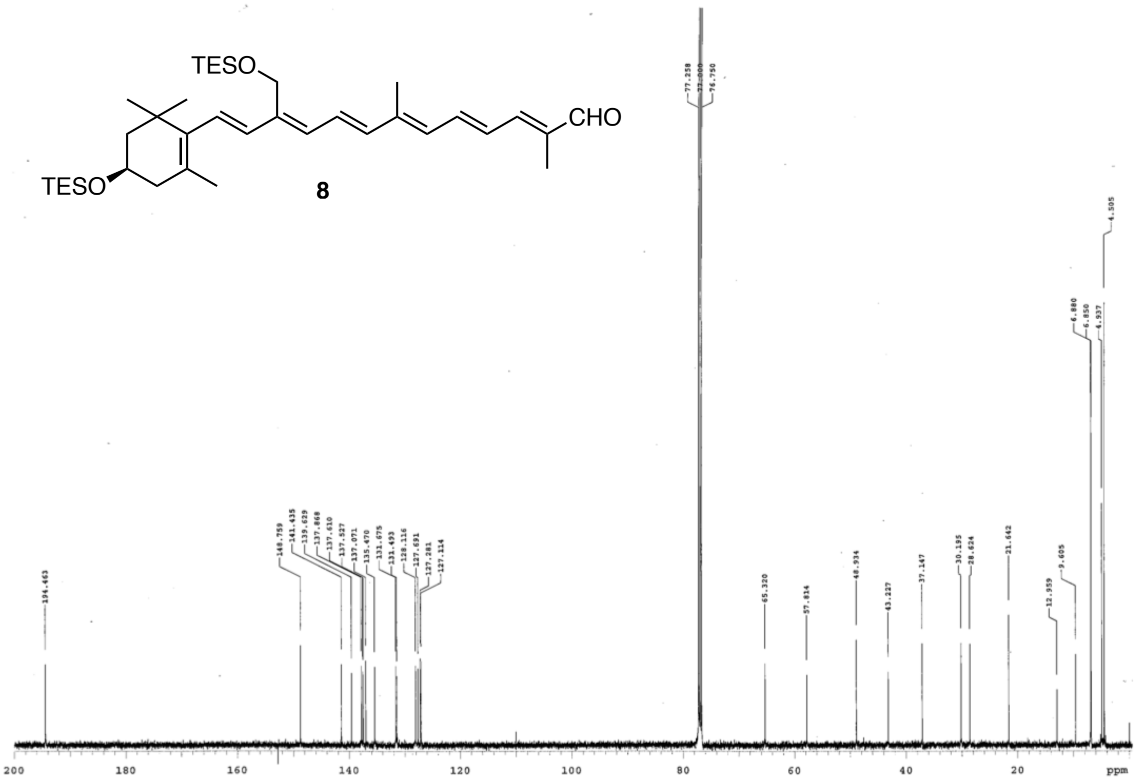

Figure S14. ^{13}C-NMR spectrum ($CDCl_3$, 125 MHz) of compound **8**

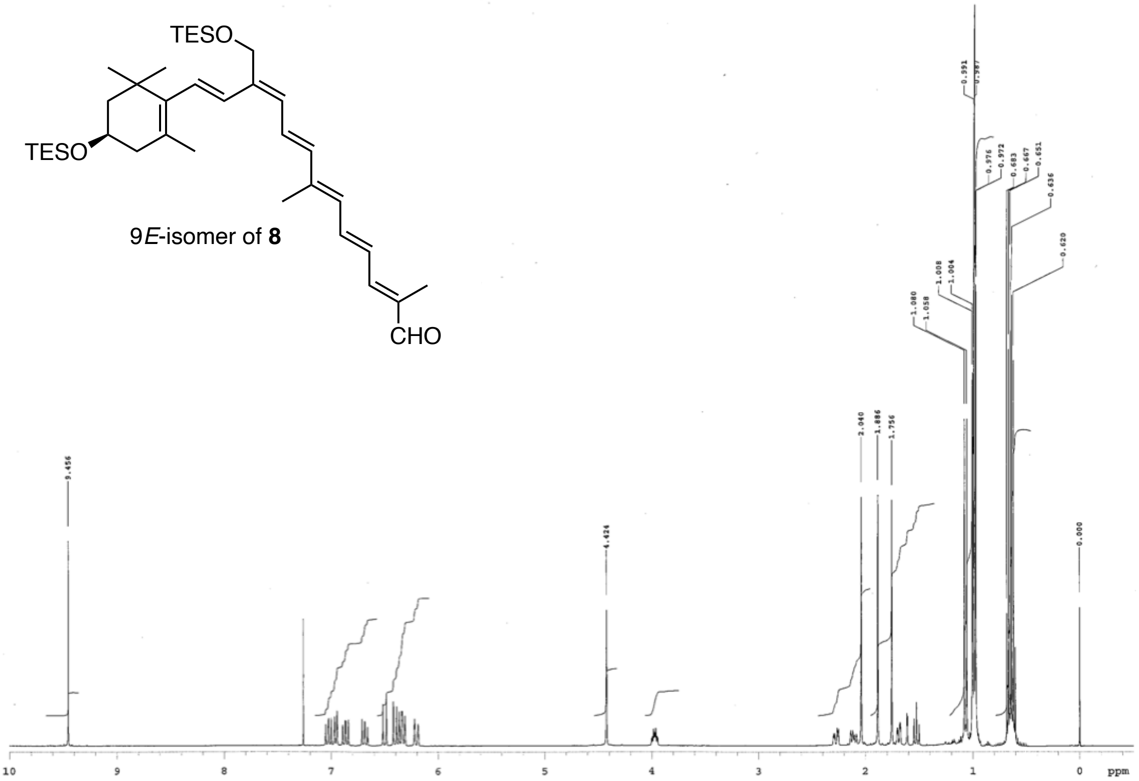

Figure S15. ^{1}H-NMR spectrum ($CDCl_3$, 500 MHz) of 9*E*-isomer of compound **8**

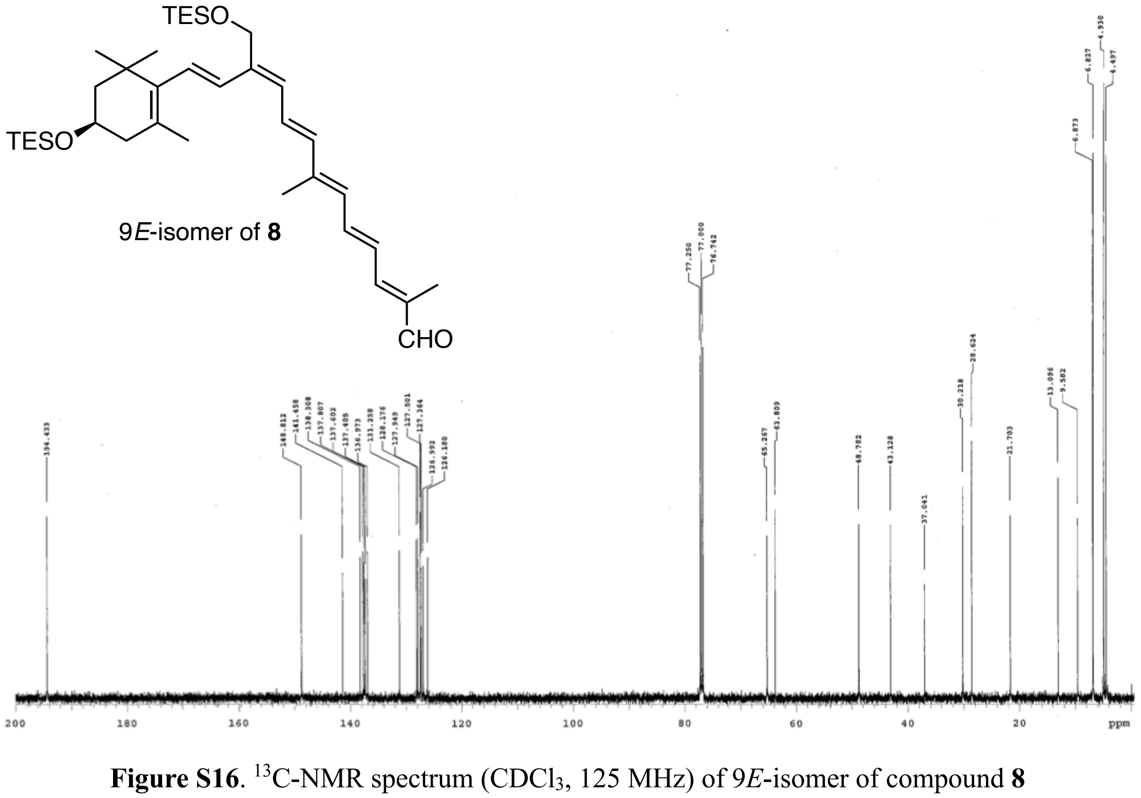

Figure S16. ^{13}C-NMR spectrum ($CDCl_3$, 125 MHz) of 9*E*-isomer of compound **8**

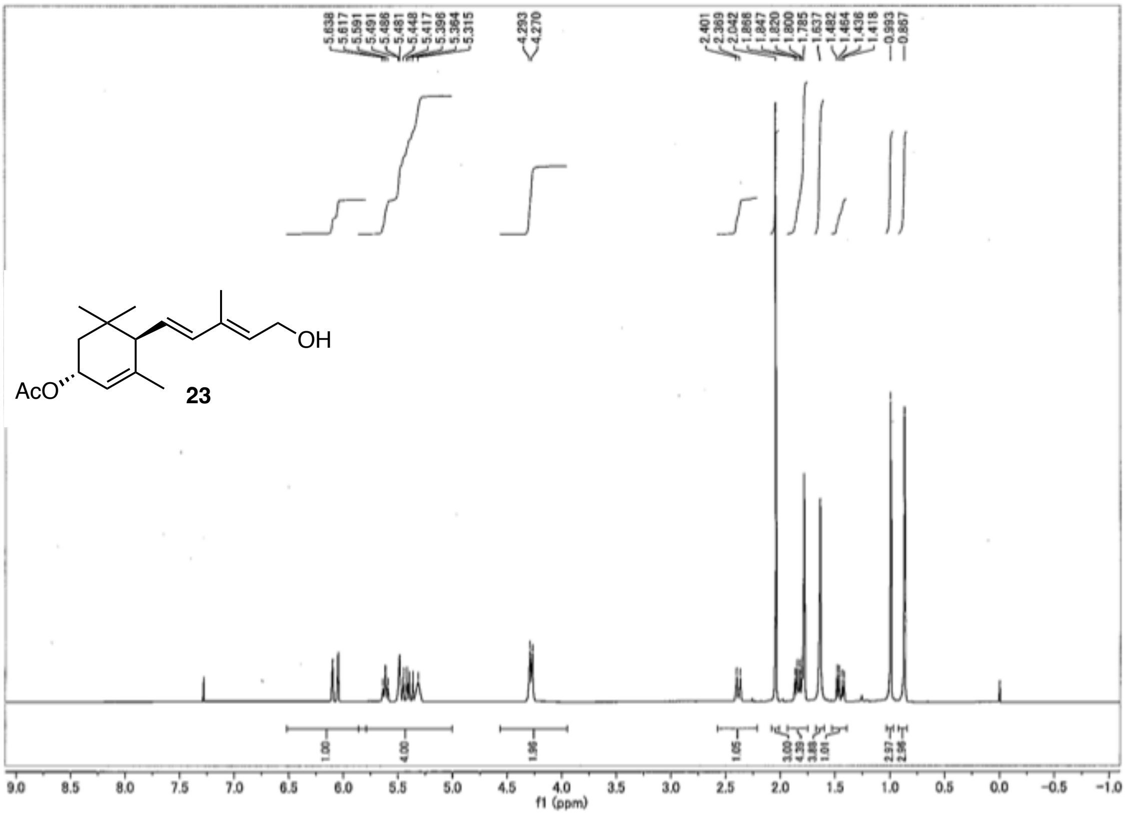

Figure S17. ^{1}H-NMR spectrum ($CDCl_3$, 300 MHz) of compound **23**

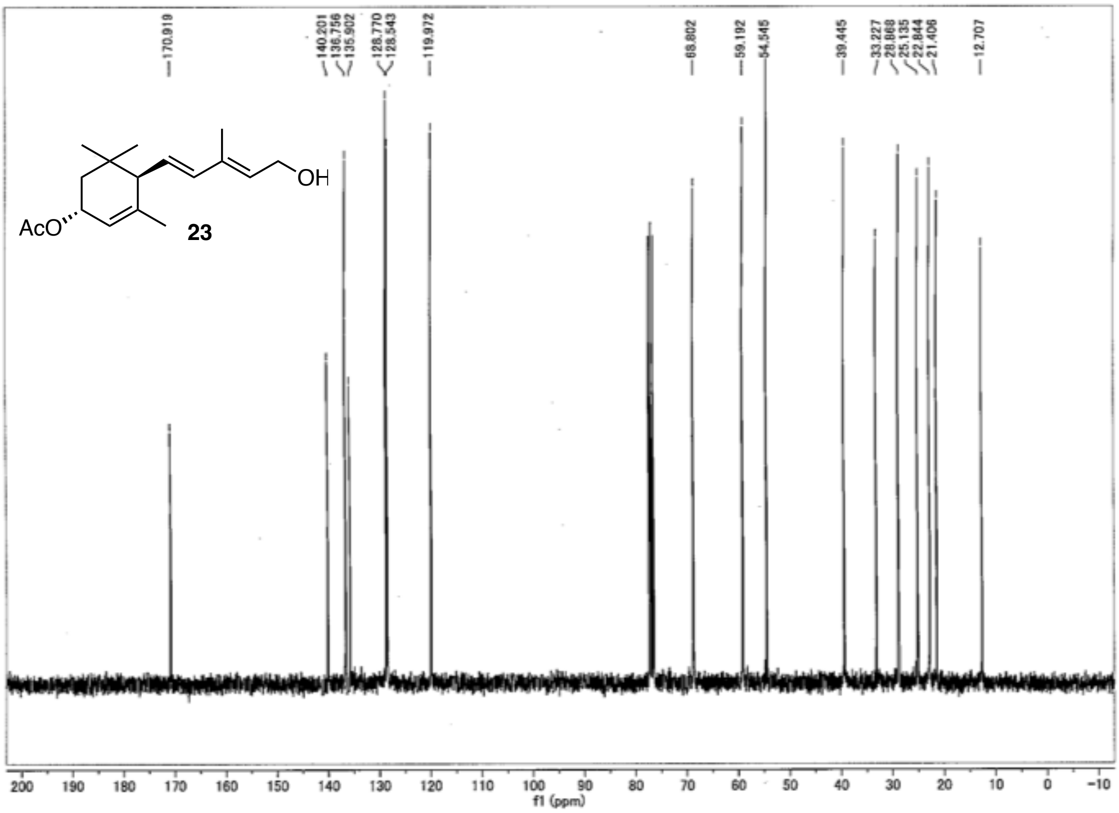

Figure S18. ^{13}C-NMR spectrum ($CDCl_3$, 75 MHz) of compound **23**

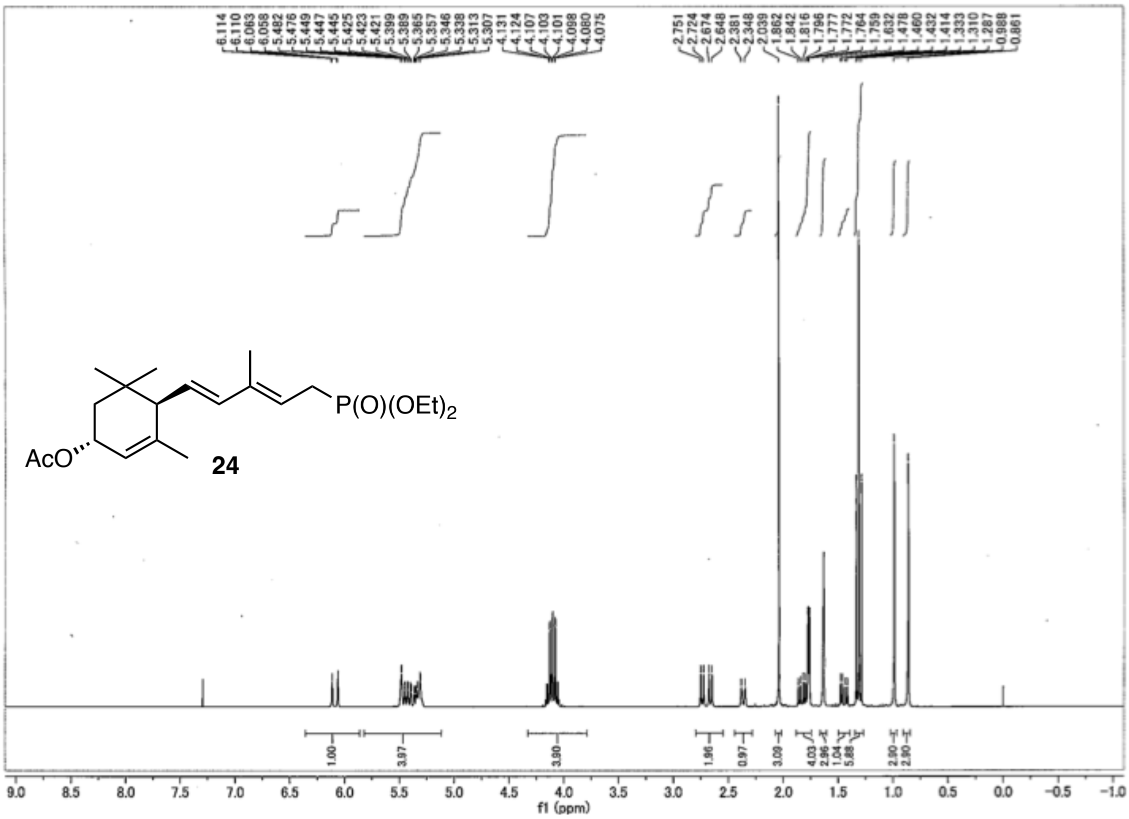

Figure S19. ^{1}H-NMR spectrum ($CDCl_3$, 300 MHz) of compound **24**

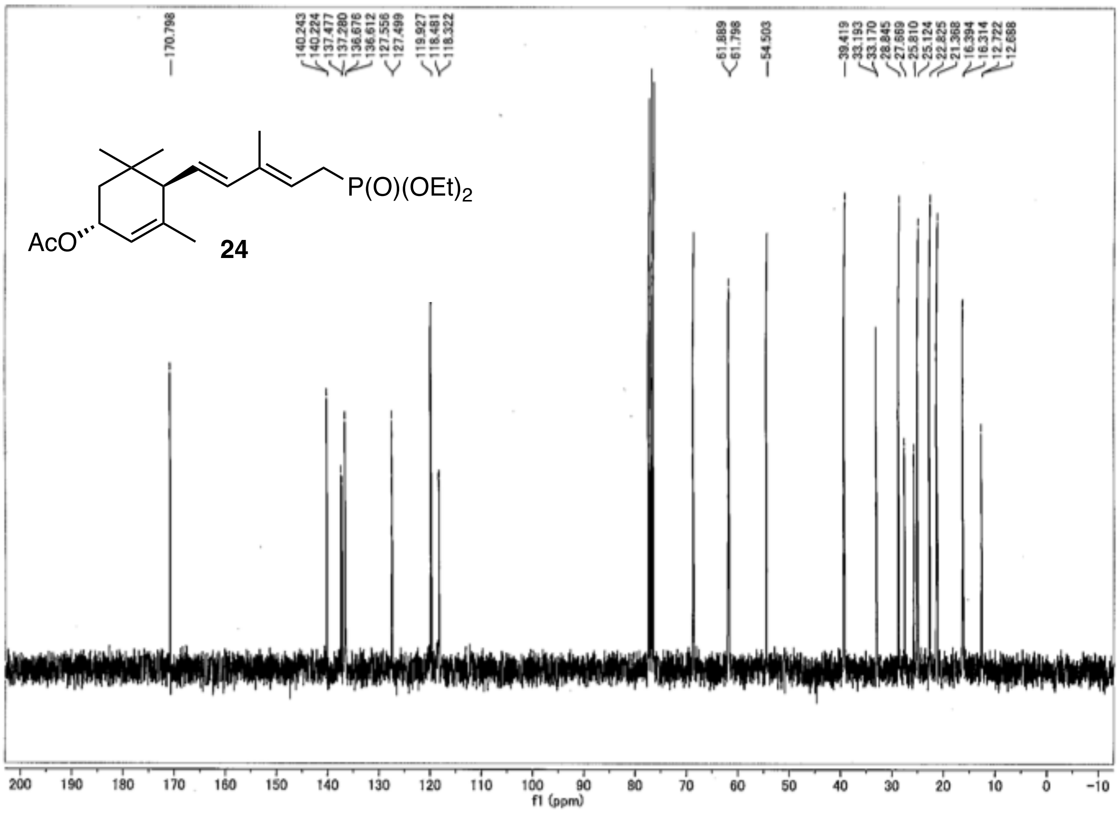

Figure S20. ^{13}C-NMR spectrum ($CDCl_3$, 75 MHz) of compound **24**

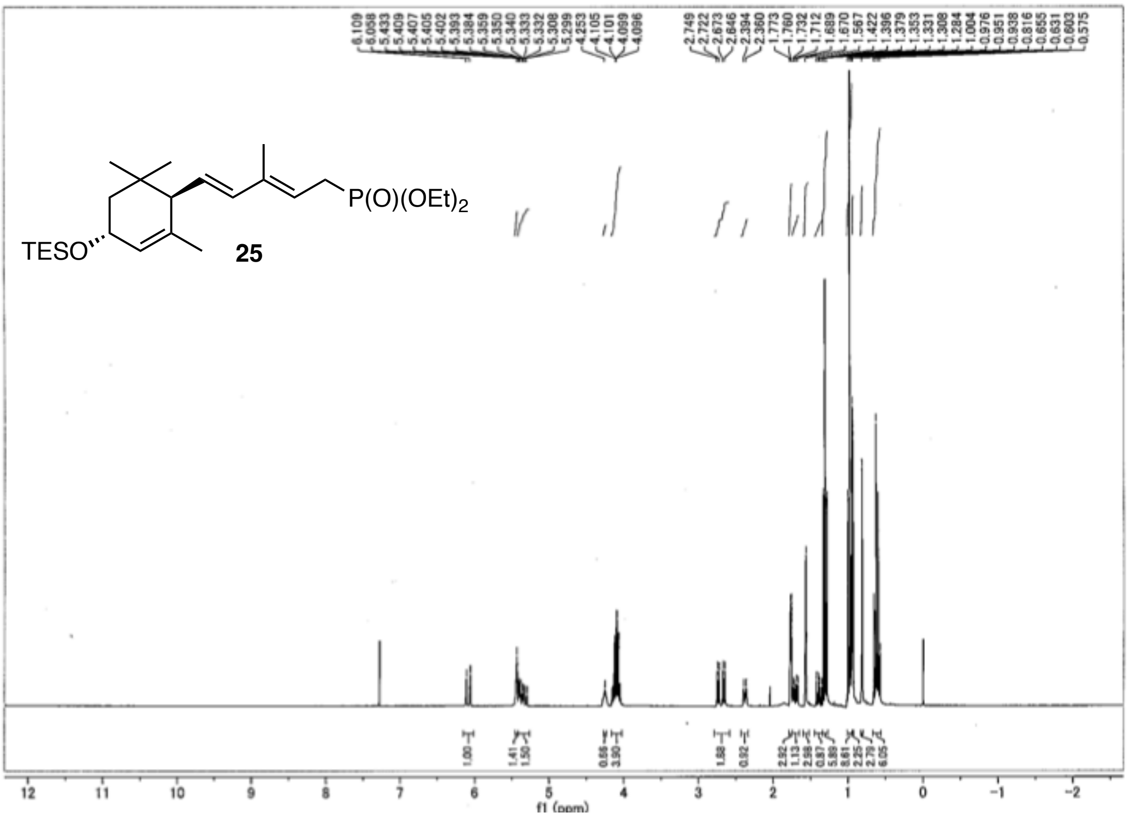

Figure S21. ^{1}H-NMR spectrum ($CDCl_3$, 300 MHz) of compound **25**

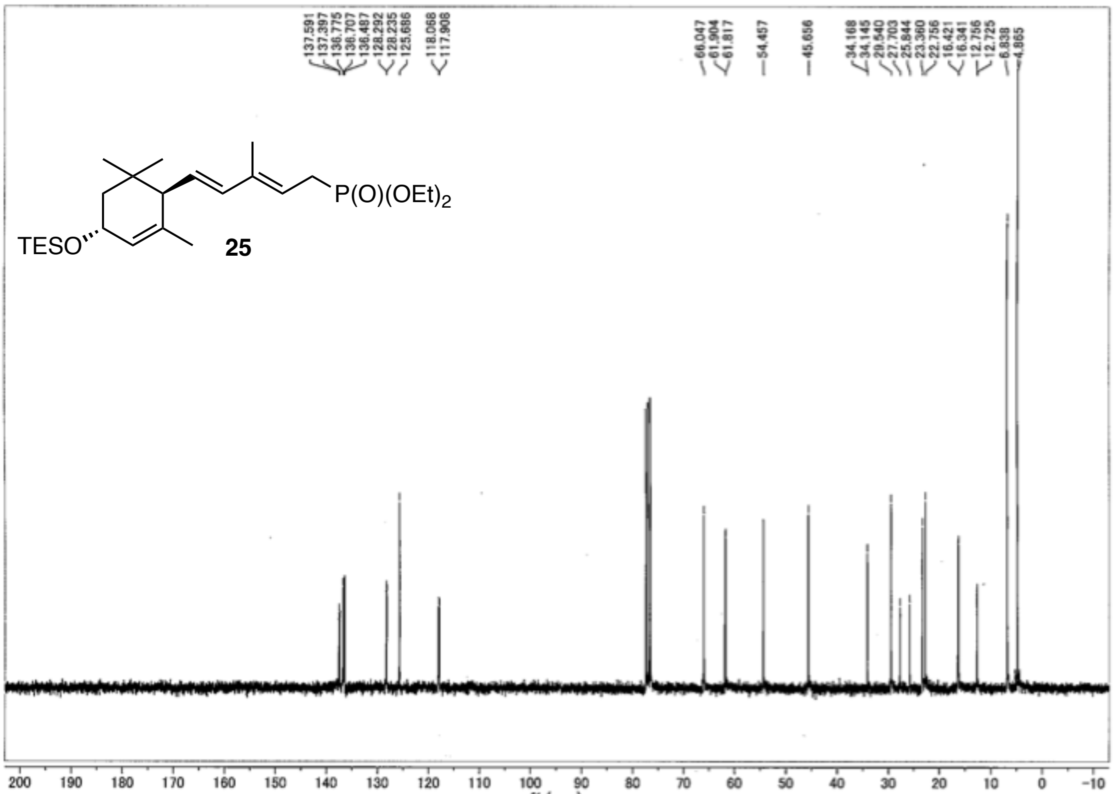

Figure S22. ^{13}C-NMR spectrum ($CDCl_3$, 75 MHz) of compound **25**

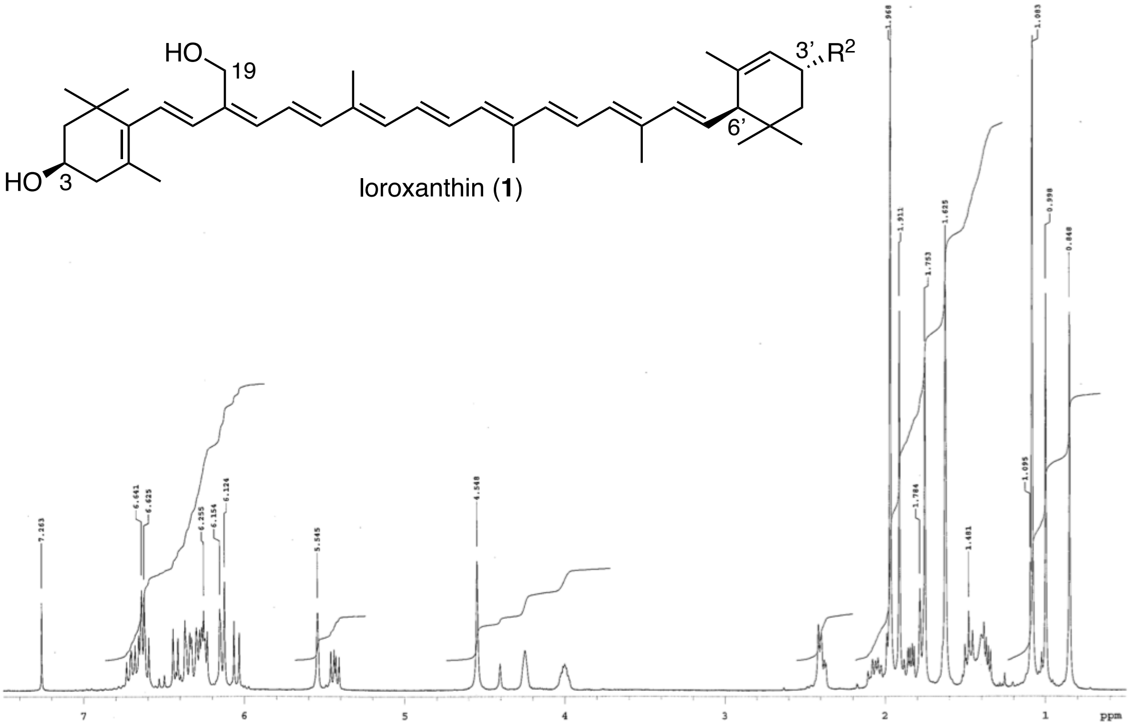

Figure S19. ^{1}H-NMR spectrum ($CDCl_3$, 500 MHz) of loroxanthin (**1**)

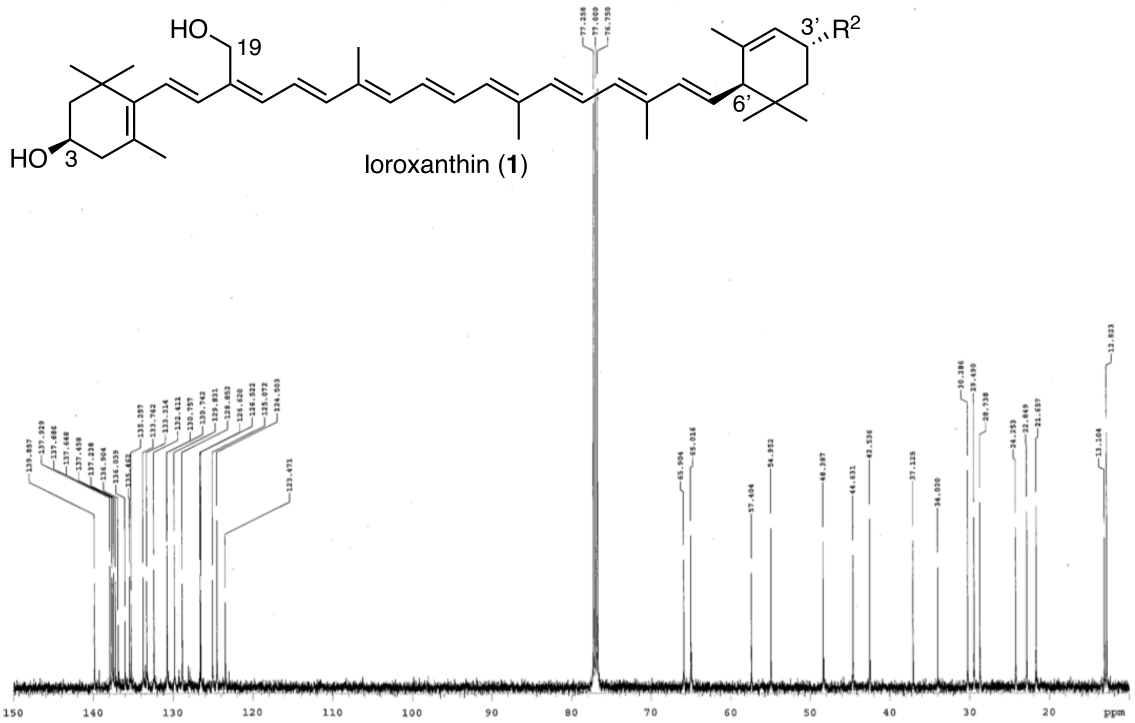

Figure S20. ^{13}C-NMR spectrum ($CDCl_3$, 125 MHz) of loroxanthin (**1**)
